# Holistic Review of Applicants by the United States Postgraduate Training Programs Is Not Associated With the Percentage of Female Residents

**DOI:** 10.7759/cureus.58002

**Published:** 2024-04-10

**Authors:** Kaitlin M Bowers, Mary E Gresham, Vishnu Mudrakola, Jeremy Shapiro, Richelle J Cooper, Jestin N Carlson, Dhimitri A Nikolla

**Affiliations:** 1 Department of Emergency Medicine, Jerry M. Wallace School of Osteopathic Medicine, Campbell University, Lillington, USA; 2 Department of Emergency Medicine, Summa Health, Akron, USA; 3 Department of Emergency Medicine, Allegheny Health Network, Erie, USA; 4 Department of Emergency Medicine, University of California, Los Angeles, USA; 5 Department of Internal Medicine/Emergency Medicine, Lake Erie College of Osteopathic Medicine, Erie, USA

**Keywords:** healthcare workforce, female faculty, specialty, cross-sectional survey, diversity, postgraduate training programs, underrepresentation, female residents, residency applications, holistic review

## Abstract

Introduction

Given the underrepresentation of female physicians in most specialties and the aim of holistic review in residency applications to improve the diversity of matriculating resident physicians in the United States (US) postgraduate medical training programs, we examined the association between holistic review and female resident representation among US postgraduate training programs.

Methods

We conducted a cross-sectional survey of US postgraduate training programs to inquire about their use of holistic review for resident applications (independent variable). The primary outcome was the percentage of female residents in each program, which was obtained along with other program-level characteristics from the Fellowship and Residency Electronic Interactive Database Access (FREIDA) catalog in April 2023. We limited the analysis to the 10 specialties with the most training spots in 2022, including anesthesiology, emergency medicine, family medicine, internal medicine, neurology, obstetrics and gynecology, orthopedic surgery, pediatrics, psychiatry, and surgery (general). We also examined the interactions between holistic review and specialty and the percentage of female faculty using model comparison and simple slopes analyses.

Results

Of the 3,364 total programs surveyed from the 10 specialties, 222 (6.6%) responded. Responders and nonresponders had similar program-level characteristics, including program type (e.g., university, community), specialty, and reported minimum board examination scores. Of the 222 responders, 179 (80.6%) reported performing holistic review. The percentage of female residents was 49.0% (interquartile range 37.5 to 66.7) in the no holistic review group and 47.8% (35.4 to 65.0) in the holistic review group (median difference 0.9%, 95% confidence interval -6.7 to 8.3). Furthermore, there was no evidence of interaction between holistic review and either the specialty or the percentage of female faculty on the outcome of the percentage of female residents.

Conclusions

Holistic review of residency applications in this limited sample of US postgraduate training programs was not associated with the percentage of female residents. The role of holistic review in addressing the imbalance of male and female physicians in the healthcare workforce, particularly between specialties, remains unknown.

## Introduction

Diversity in the healthcare workforce is vital to mitigating health disparities. A major barrier to diversity in medicine is the disproportionate number of female physicians compared to female patients. While half of the patients are female, only about a third of physicians are female [1,2]. This discordance is problematic because it may compromise the care of patients. For example, care from male physicians to female patients has been associated with worse outcomes among surgical and myocardial infarction patients [3,4]. Additionally, elderly patients being treated by female internists had lower 30-day mortality and readmission rates than those treated by their male counterparts [5]. These outcome differences may be due to practice differences between male and female physicians. Prior work has shown that female physicians are more likely to follow clinical guidelines [6,7], focus on patient counseling/communication [8], and emphasize preventative care [9-15]. However, female representation among United States (US) physicians varies greatly across specialties [2].

Female physicians are underrepresented in most specialties. Among US physicians in 2021, 39 of 49 (79.6%) medical specialties are composed of <50% female physicians [2]. Furthermore, female physicians are severely underrepresented in many specialties, such as orthopedic surgery, where only 1,090 of 18,464 (5.9%) surgeons are female [2]. This variability in female physician representation across specialties begins at the postgraduate training level. For example, only 13.7% of residents in orthopedic surgery are female [16]. Interventions intended to diminish the sex and/or gender disparity across specialties in US postgraduate training programs may offer an opportunity to ameliorate differences in patient outcomes associated with physician-patient sex discordance.

Holistic review of residency applicants may affect the balance of matriculating male and female physicians into postgraduate training programs. Holistic review is a resident selection process and/or strategy with the goal of evaluating the "whole" applicant (i.e., experiences and attributes in addition to academic performance) without disproportionately prioritizing any one factor [17]. Holistic review is intended to improve the diversity of matriculating residents [18,19], and it may employ a weighted scoring system to balance academic and nonacademic factors [20]. By implementing holistic review processes in residency applicant selection, postgraduate training programs have improved the diversity of trainees at their sites [20-22]. However, the relationship between holistic review and female resident representation has not been explored.

Objective

Our primary aim was to examine program characteristics associated with the self-reported performance of holistic review of each resident application, particularly the percentage of female residents. We hypothesized that holistic review would not be associated with the program-level percentage of female residents across specialties, but it may be associated with the program-level percentage of female residents within specialties with an overrepresentation of one sex. The second aim was to explore the interaction between holistic review and medical specialty on the outcome of the program-level percentage of female residents. We hypothesized that holistic review would modify the association between specialty and the percentage of female residents. The third aim was to explore the interaction between holistic review and the program-level percentage of female faculty on the outcome of the percentage of female residents. We hypothesized that a holistic review would modify the association between the percentages of female faculty and residents.

## Materials and methods

Study design and data collection

To obtain program-reported use of holistic review of resident applications, we performed a cross-sectional survey of the Accreditation Council for Graduate Medical Education (ACGME)-approved postgraduate training programs (Appendix). We then linked these responses with program characteristics reported in the catalog of the Fellowship and Residency Electronic Interactive Database Access (FREIDA) [23]. FREIDA data were obtained through a data licensing agreement with the American Medical Association. Among the programs that responded to our survey, the FREIDA data of each program were updated between June 2022 and December 2022, with three (1.4%) programs missing the last update dates. The FREIDA database does not clarify whether male/female refers to the sex (i.e., male, female, intersex) or gender (e.g., man, woman, transgender, nonbinary) of the physicians. However, the percentages of male and female resident physicians are reported without a variable for intersex. Therefore, we used the terms male and female to describe our results. Our results are reported in accordance with the Checklist for Reporting Results of Internet E-Surveys (checklist presented in the Appendix) [24].

Sample characteristics

We surveyed program directors from all ACGME-approved programs in the 10 specialties with the highest number of training positions in 2022, constituting 78.5% of all training spots in 2022 [25]. These specialties included anesthesiology, emergency medicine, family medicine, internal medicine, neurology, obstetrics and gynecology, orthopedic surgery, pediatrics, psychiatry, and surgery (general), which represent a diverse sex distribution from approximately 13% female residents in orthopedic surgery to approximately 81% in obstetrics and gynecology [1,16]. We excluded transitional and preliminary programs as well as specialties with fewer training spots since these programs may, on average, have fewer applicants, lowering the work barriers to performing a holistic review of every application compared to programs with more applicants [26].

Survey administration

The survey was administered using a web-based tool (Jotform Inc., San Francisco, California). The investigators tested the web address functionality, but given the simplicity of the three-question survey, it was not formally tested in, for example, a pilot study. The survey was emailed to program directors as an “open survey” thrice from April to June 2023. Program director emails were obtained from the ACGME website [27]. If the email was undeliverable or the individual responded as being no longer affiliated with the program, we searched for the missing program director emails on the individual program websites and by web search. The participation and completion rates were not measured because we could measure the true response rate, given that we surveyed all programs within the included specialties. IP addresses and cookies were not used to identify specific users or assign user identifiers since we collected unique ACGME IDs for each program response. The time to complete the survey was not measured.

Survey preparation and ethical considerations

On the first webpage of the survey, participants were given the expected time to complete the survey (i.e., two minutes), the study purpose, the principal investigator’s contact information, and what data would be requested. They were informed that responding was completely voluntary, their responses would be linked to program characteristics, there were no direct benefits from participating, and the results would be reported in aggregate to ensure anonymity. Informed consent was assumed by participating in the survey, and the study protocol was reviewed and given exempt status by the Allegheny Health Network Research Institute Institutional Review Board (#2023-037).

On the second webpage of the survey, participants were presented with three questions. First, we asked for the program’s ACGME ID to link the responses to the FREIDA data. Next, we asked if the program performed a holistic review of every trainee application. We provided our definition of holistic review: holistic review is a process and/or strategy of evaluating the “whole” applicant (i.e., experiences and attributes in addition to academic performance) without disproportionately prioritizing any one factor. Lastly, we asked if the program would allow us to contact them to complete any missing program data from FREIDA (Appendix). The questions were not randomized, and all three questions were on the second webpage. Respondents could not change their answers once the survey was submitted.

Outcome and variables

Our primary outcome was the three-year average of the percentage of female residents reported in FREIDA, while our independent variable was the program-reported holistic review reported from the survey described above. Other variables examined from FREIDA included program type (e.g., university, community), specialty, reported minimum board examination score (either the US Medical Licensing Examination (USMLE) or Comprehensive Osteopathic Medical Licensing Examination (COMLEX-USA)), required USMLE for osteopathic applicants, visa acceptance (J1, H1B, F1), osteopathic recognition, number of first-year positions, the percentage of female faculty, and the percentage of osteopathic and international medical graduate (IMG) residents. The collection and coding of each variable are detailed in the Appendix.

Statistical analysis

For our primary aim, we examined the difference in the percentage of female residents between programs reporting and not reporting the use of holistic review. However, since the baseline percentage of female residents varies by specialty [16], we conducted sensitivity analyses exploring the association between holistic review and the percentage of female residents accounting for specialty-level differences. First, we examined the association between holistic review and the difference between each program’s proportion of female residents and the median for the program’s specialty. However, since some specialties match substantially more females and some substantially more males [1,16], we also examined the association between holistic review and the difference between each program’s percentage of female residents and the percentage of females applying overall in the National Resident Matching Program (NRMP) match in 2022 (20,024 of 40,735 applicants or 49.2%) [28]. Lastly, we performed subgroup analyses examining the association between holistic review and the percentage of female residents in specialties with less than 40% female residents (emergency medicine, anesthesiology, and orthopedic surgery) and greater than 60% female residents (obstetrics and gynecology and pediatrics).

For our second aim, we explored the interaction between holistic review and specialty in terms of the outcome of the percentage of female residents. Using linear regression, we created an interaction model with the independent variable of specialty, the dependent variable of the percentage of female residents, holistic review as a covariate, and the interaction terms between specialty and holistic review (full model). The reduced model excluded the interaction terms. Subsequently, we used the likelihood ratio test to test for the interaction.

For our third aim, we explored the interaction between holistic review and the percentage of female faculty on the outcome of the percentage of female residents. First, we tested for the presence of an interaction using a simple slopes analysis with an interaction plot. The interaction plot is a scatter plot of the independent and dependent variables (percentage of female faculty and residents) with two regression lines comparing programs that reported using and not using holistic review. To further test for the interaction, we created an interaction model with the independent variable of the percentage of female faculty, the dependent variable of the percentage of female residents, holistic review as a covariate, and the interaction term between the percentage of female faculty and holistic review (full model), using linear regression. The reduced model excluded the interaction term. Subsequently, we used the likelihood ratio test to test for the interaction.

We checked continuous variables for normality using the Shapiro-Wilk test and homoscedasticity using the studentized Breusch-Pagan test since these are assumptions for linear regression. Categorical data were reported as counts with percentages, while continuous data were presented as means with standard deviations (SD) or medians with interquartile ranges (IQR) depending on normality. Percent, mean, and median differences between the holistic review and no holistic review groups were determined by the two-sample test for equality of proportions with continuity correction, the t-test, or the Wilcoxon rank sum test. For the outcomes, a confidence interval (CI) excluding zero or a p-value of <0.05 was considered significant. The analysis was performed with R version 4.3.0 (R Foundation for Statistical Computing, Vienna, Austria).

Missingness

Of the unique 222 programs that responded, 19 (8.6%) had missing values for our primary outcome: the percentage of female residents. Therefore, we performed multiple imputations of the study dataset and repeated all the above analyses. We used random forest multiple imputation with 10 iterations and 100 trees, including the abovementioned variables. A complete list of predictor variables used and additional details on the multiple imputation methods are presented in the Appendix. All surveys were complete. Three programs had duplicate responses without discrepancies between items, so the additional responses were excluded.

## Results

Respondent characteristics

Of the 3,364 total programs surveyed from the 10 specialties, 18 of 164 (11.0%) anesthesiology programs, 35 of 283 (12.4%) emergency medicine programs, 48 of 743 (6.5%) family medicine programs, 25 of 617 (4.1%) internal medicine programs, 13 of 177 (7.3%) neurology programs, 18 of 298 (6.0%) obstetrics and gynecology programs, 9 of 208 (4.3%) orthopedic surgery programs, 21 of 216 (9.7%) pediatrics programs, 18 of 302 (6.0%) psychiatry programs, and 17 of 356 (4.8%) surgery (general) programs responded (Table 1). The greatest number of respondents were from family medicine (48 of 222 or 21.6%) and community-based university-affiliated hospitals (96 of 222 or 43.2%) (Table 1). The respondents and nonrespondents were similar across program characteristics (Table 1).

**Table 1:** Responders and nonresponders ^a^Mean, median, or percent difference depending on data type and distribution if continuous CI, confidence interval; DO, doctor of osteopathic medicine; IMG, international medical graduate; IQR, interquartile range; SD, standard deviation

Characteristics	Responded to Survey		Difference (95% CI)^a^
	No	Yes	
Total, n	3,142	222	
Program type, n (%)			
Community-based university-affiliated hospital	1324 (42.1)	96 (43.2)	-1.1 (-8.1 to 5.9)
Community hospital	696 (22.2)	44 (19.8)	2.3 (-3.4 to 8)
Military-based	69 (2.2)	1 (0.5)	1.7 (0.5 to 3)
University hospital	1037 (33)	80 (36)	-3 (-9.8 to 3.7)
Other	16 (0.5)	1 (0.5)	0.1 (-0.9 to 1)
Specialty, n (%)			
Anesthesiology	146 (4.6)	18 (8.1)	-3.5 (-7.4 to 0.4)
Emergency medicine	248 (7.9)	35 (15.8)	-7.9 (-13 to -2.7)
Family medicine	695 (22.1)	48 (21.6)	0.5 (-5.3 to 6.3)
Internal medicine	592 (18.8)	25 (11.3)	7.6 (3 to 12.2)
Neurology	164 (5.2)	13 (5.9)	-0.6 (-4.1 to 2.8)
Obstetrics and gynecology	280 (8.9)	18 (8.1)	0.8 (-3.2 to 4.8)
Orthopedic surgery	199 (6.3)	9 (4.1)	2.3 (-0.7 to 5.3)
Pediatrics	195 (6.2)	21 (9.5)	-3.3 (-7.4 to 0.9)
Psychiatry	284 (9)	18 (8.1)	0.9 (-3 to 4.9)
Surgery (general)	339 (10.8)	17 (7.7)	3.1 (-0.8 to 7)
Minimum board examination score, n (%)			
Yes	2221 (70.7)	164 (73.9)	-3.2 (-9.4 to 3)
USMLE required for DOs, n (%)			
Yes	734 (23.4)	42 (18.9)	4.4 (-1.2 to 10)
Missing	156 (5)	8 (3.6)	1.4 (-1.4 to 4.2)
J1 visa sponsored, n (%)			
Yes	2068 (65.8)	152 (68.5)	-2.7 (-9.2 to 3.9)
Missing	104 (3.3)	4 (1.8)	1.5 (-0.6 to 3.6)
H1B visa sponsored, n (%)			
Yes	589 (18.7)	40 (18)	0.7 (-4.7 to 6.2)
Missing	145 (4.6)	8 (3.6)	1 (-1.8 to 3.8)
F1 visa sponsored, n (%)			
Yes	398 (12.7)	31 (14)	-1.3 (-6.2 to 3.6)
Missing	160 (5.1)	8 (3.6)	1.5 (-1.3 to 4.3)
Osteopathic recognition, n (%)			
Yes	198 (6.3)	21 (9.5)	-3.2 (-7.3 to 1)
Missing	351 (11.2)	21 (9.5)	1.7 (-2.5 to 6)
First-year positions, median (IQR)	8 (5 to 12)	8 (6 to 12)	0 (-1 to 0)
Missing, n (%)	2 (0.1)	0 (0.0)	0.06 (-0.09 to 0.22)
Percent DO residents, median (IQR)	15.4 (1.8 to 40)	18.8 (4.3 to 44.4)	-0.9 (-4.3 to 0.0)
Missing, n (%)	227 (7.2)	19 (8.6)	-1.3 (-5.4 to 2.7)
Percent IMG residents, median (IQR)	9.1 (0.0 to 39.5)	8.3 (0.0 to 29.7)	0.0 (0.0 to 2.2)
Missing, n (%)	227 (7.2)	19 (8.6)	-1.3 (-5.4 to 2.7)
Percent female residents, median (IQR)	47.4 (35.5 to 61.5)	47.8 (35.5 to 66.4)	-1.1 (-4.2 to 1.9)
Missing, n (%)	227 (7.2)	19 (8.6)	-1.3 (-5.4 to 2.7)
Percent female faculty, mean (SD)	40.7 (20.7)	44.4 (20.3)	-3.7 (-6.6 to -0.8)
Missing, n (%)	336 (10.7)	16 (7.2)	3.5 (-0.3 to 7.3)

Descriptive results

Of the 222 respondents, 179 (80.6%) programs reported performing holistic review. Programs that reported not performing holistic review vs. performing holistic review were similar across all examined variables, including program type, specialty, USMLE requirement for DO applicants, number of first-year positions, and the proportions of female faculty as well as DO and IMG residents (Table 2). Minimum board examination scores were reported for 32 of 43 (74.4%) programs not performing holistic review and 132 of 179 (73.7%) performing holistic review (percent difference 0.7%, 95% CI -14.5 to 15.9) (Table 2). Missingness was <10% for all the study variables with missing values (Table 1) but was balanced between programs performing and not performing holistic review (Table 2).

**Table 2:** Program characteristics by performance of holistic review ^a^Mean, median, or percent difference depending on data type and distribution if continuous CI, confidence interval; DO, doctor of osteopathic medicine; IMG, international medical graduate; IQR, interquartile range; SD, standard deviation

Characteristic	Holistic Review		Difference (95% CI)^a^
	No	Yes	
Total, n	43	179	
Program type, n (%)			
Community-based university-affiliated hospital	17 (39.5)	79 (44.1)	-4.6 (-22.4 to 13.2)
Community hospital	11 (25.6)	33 (18.4)	7.1 (-8.5 to 22.8)
Military-based	0 (0)	1 (0.6)	-0.6 (-2.2 to 1.1)
University hospital	15 (34.9)	65 (36.3)	-1.4 (-18.8 to 15.9)
Other	0 (0)	1 (0.6)	-0.6 (-2.2 to 1.1)
Specialty, n (%)			
Anesthesiology	5 (11.6)	13 (7.3)	4.4 (-7.4 to 16.1)
Emergency medicine	5 (11.6)	30 (16.8)	-5.1 (-17.6 to 7.3)
Family medicine	7 (16.3)	41 (22.9)	-6.6 (-20.7 to 7.5)
Internal medicine	8 (18.6)	17 (9.5)	9.1 (-4.7 to 22.9)
Neurology	2 (4.7)	11 (6.1)	-1.5 (-10.1 to 7.2)
Obstetrics and gynecology	4 (9.3)	14 (7.8)	1.5 (-9.5 to 12.5)
Orthopedic surgery	2 (4.7)	7 (3.9)	0.7 (-6.9 to 8.4)
Pediatrics	4 (9.3)	17 (9.5)	-0.2 (-10.1 to 9.7)
Psychiatry	3 (7)	15 (8.4)	-1.4 (-11.4 to 8.6)
Surgery (general)	3 (7)	14 (7.8)	-0.8 (-10.3 to 8.6)
Minimum board examination score, n (%)			
Yes	32 (74.4)	132 (73.7)	0.7 (-14.5 to 15.9)
USMLE required for DOs, n (%)			
Yes	11 (25.6)	31 (17.3)	8.3 (-7.3 to 23.9)
Missing	2 (4.7)	6 (3.4)	1.3 (-6.8 to 9.4)
J1 visa sponsored, n (%)			
Yes	29 (67.4)	123 (68.7)	-1.3 (-18.1 to 15.6)
Missing	0 (0)	4 (2.2)	-2.2 (-5.8 to 1.4)
H1B visa sponsored, n (%)			
Yes	9 (20.9)	31 (17.3)	3.6 (-11.2 to 18.4)
Missing	0 (0)	8 (4.5)	-4.5 (-8.9 to 0)
F1 visa sponsored, n (%)			
Yes	7 (16.3)	24 (13.4)	2.9 (-10.7 to 16.4)
Missing	0 (0)	8 (4.5)	-4.5 (-8.9 to 0)
Osteopathic recognition, n (%)			
Yes	2 (4.7)	19 (10.6)	-6 (-15.2 to 3.2)
Missing	3 (7)	18 (10.1)	-3.1 (-13.3 to 7.2)
First-year positions, median (IQR)	8 (6 to 13)	8 (6 to 12)	0 (-1 to 2)
Percent DO residents, median (IQR)	15.2 (0 to 45.5)	20 (4.5 to 43.5)	-1.5 (-10.1 to 4.2)
Missing, n (%)	4 (9.3)	15 (8.4)	0.9 (-9.6 to 11.4)
Percent IMG residents, median (IQR)	6.1 (0 to 22.5)	9.1 (0 to 30.1)	0 (-4.7 to 2.1)
Missing, n (%)	4 (9.3)	15 (8.4)	0.9 (-9.6 to 11.4)
Percent female faculty, mean (SD)	43.6 (20.4)	44.6 (20.4)	-1.1 (-8.1 to 6)
Missing, n (%)	1 (2.3)	15 (8.4)	-6.1 (-13.6 to 1.5)

Aim 1

The percentage of female residents was 49.0% (IQR 37.5 to 66.7) in the no holistic review group and 47.8% (35.4 to 65.0) in the holistic review group (median difference 0.9%, 95% CI -6.7 to 8.3). There was no difference between each program’s percentage of female residents and the median for the program’s specialty without and with holistic review, 1.2% (SD 15.7) vs. 0.1% (SD 12.9), respectively (mean difference 1.0%, 95% CI -4.4 to 6.5). There was no difference between each program’s percentage of female residents and the percentage of females applying overall without and with holistic review, 14.3% (IQR 5.4 to 25.8) vs. 14.9% (6.6 to 25.7), respectively (median difference 0.0%, 95% CI -4.2 to 4.3). Lastly, among programs with less than 40% female residents (emergency medicine, anesthesiology, and orthopedic surgery), the percentage of female residents was 40.9% (SD 14.9) vs. 33.8% (SD 15.1) without and with holistic review (mean difference 7.2%, 95% CI -3.5 to 17.8). Additionally, among programs with greater than 60% female residents (obstetrics and gynecology and pediatrics), the percentage of female residents was 76.8% (SD 6.3) vs. 78.3% (SD 11.9) without and with holistic review (mean difference -1.5%, 95% CI -7.9 to 4.9) (Table 3). Similar results were obtained for the primary outcome and each sensitivity analysis after the multiple imputation of missing values for the percentage of female faculty and residents (Table 4).

**Table 3:** Outcomes ^a^The percentage of female residents was missing for 4 of 43 programs (9.3%) in the no holistic review group and 15 of 182 (8.4%) in the holistic review group (percent difference of 0.9%, 95% CI -9.6 to 11.4) ^b^Mean or median difference depending on the distribution ^c^These subgroups are the specialties with less than 40% female residents (emergency medicine, anesthesiology, and orthopedic surgery) and greater than 60% female residents (obstetrics and gynecology and pediatrics) AN, anesthesiology; CI, confidence interval; DO, doctor of osteopathic medicine; EM, emergency medicine; IMG, international medical graduate; IQR, interquartile range; OB, obstetrics and gynecology; Ortho, orthopedic surgery; Ped, pediatrics; SD, standard deviation

			Holistic Review		
Analysis	Female residents	N^a^	No	Yes	Difference (95% CI)^b^
Primary	Program % female, median (IQR)	203	49.0 (37.5 to 66.7)	47.8 (35.4 to 65.0)	0.9 (-6.7 to 8.3)
Sensitivity	Difference between program % female and median % of specialty, mean (SD)	203	1.2 (15.7)	0.1 (12.9)	1.0 (-4.4 to 6.5)
	Difference between program % female and overall % female applicants, median (IQR)	203	14.3 (5.4 to 25.8)	14.9 (6.6 to 25.7)	0.0 (-4.2 to 4.3)
Subgroup	Program % female (OB and Ped subgroup), mean (SD)^c^	39	76.8 (6.3)	78.3 (11.9)	-1.5 (-7.9 to 4.9)
	Program % female (EM, AN, and Ortho subgroup), mean (SD)^c^	57	40.9 (14.9)	33.8 (15.1)	7.2 (-3.5 to 17.8)

**Table 4:** Outcomes after multiple imputation ^a^Mean or median difference depending on the distribution ^b^These subgroups are the specialties with less than 40% female residents (emergency medicine, anesthesiology, and orthopedic surgery) and greater than 60% female residents (obstetrics and gynecology and pediatrics) AN, anesthesiology; CI, confidence interval; DO, doctor of osteopathic medicine; EM, emergency medicine; IMG, international medical graduate; IQR, interquartile range; OB, obstetrics and gynecology; Ortho, orthopedic surgery; Ped, pediatrics; SD, standard deviation

			Holistic Review		
Analysis	Female residents	N	No	Yes	Difference (95% CI)^a^
Primary	Program % female, median (IQR)	222	47.4 (37.5 to 65.0)	47.5 (35.8 to 61.8)	0.2 (-6.4 to 6.8)
Sensitivity	Difference between program % female and median % of specialty, mean (SD)	222	1.2 (15.1)	0.0 (12.5)	1.1 (-3.8 to 6.1)
	Difference between program % female and overall % female applicants, median (IQR)	222	14.1 (4.3 to 25.0)	13.0 (5.6 to 23.6)	0.0 (-3.8 to 3.8)
Subgroup	Program % female (OB and Ped subgroup), mean (SD)^b^	39	76.8 (6.3)	78.3 (11.9)	-1.5 (-7.9 to 4.9)
	Program % female (EM, AN, and Ortho subgroup), mean (SD)^b^	62	39.8 (14.7)	34.1 (14.7)	5.7 (-4.2 to 15.7)

Aims 2 and 3

**Figure 1:**
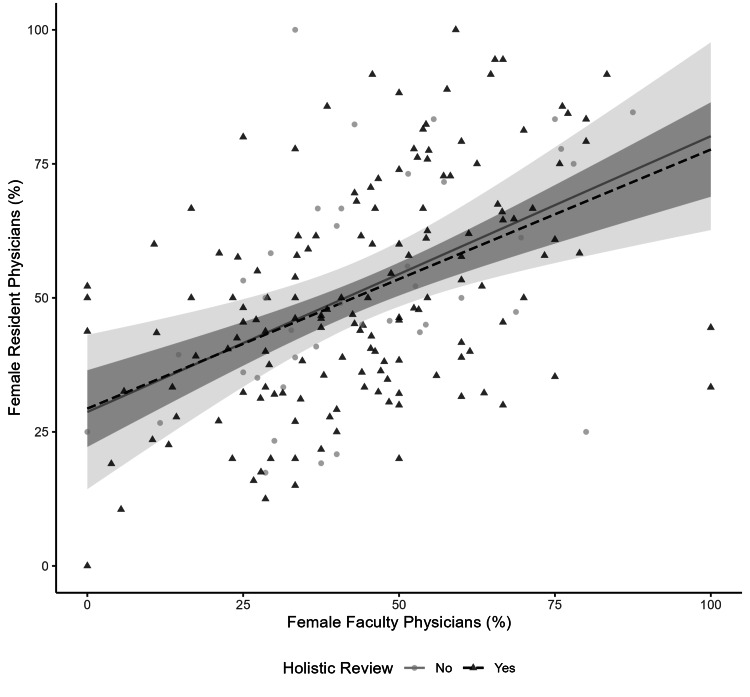
Interaction of female residents and faculty associations by holistic review The scatter plot displays each program by the percentage of female residents and faculty by holistic review. There is a best-fit line for programs reporting using and not using a holistic review of trainee applications. The best-fit lines were derived from linear regression, and the shading represents the 95% confidence intervals. Since the confidence intervals overlap, an interaction by holistic review on the association between female residents and faculty is not observed.

**Figure 2:**
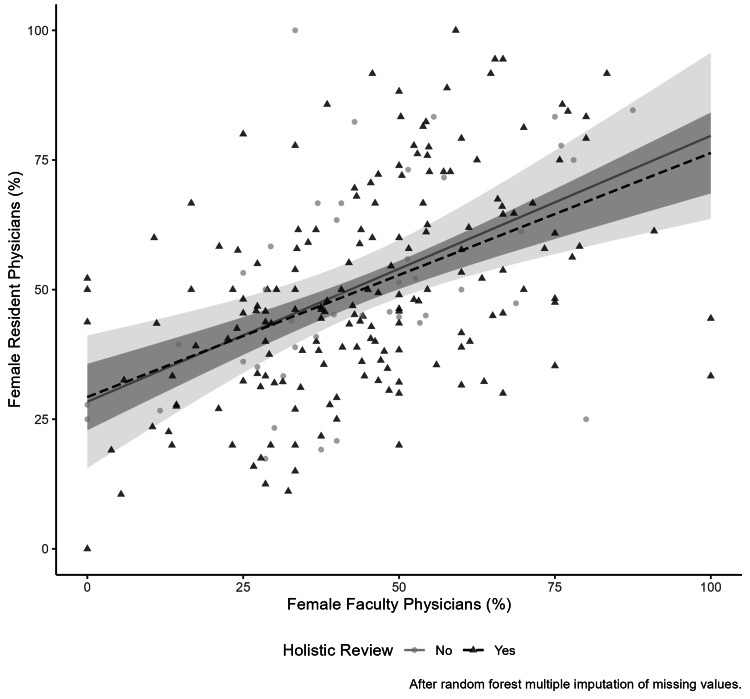
Interaction of female resident and faculty association by holistic review after multiple imputations The scatter plot displays each program by the percentage of female residents and faculty by holistic review after multiple data imputations. There is a best-fit line for programs reporting using and not using a holistic review of trainee applications. The best-fit lines were derived from linear regression, and the shading represents the 95% confidence intervals. Since the confidence intervals overlap, an interaction by holistic review on the association between female residents and faculty is not observed.

The likelihood ratio test between the full and reduced interaction models, with and without the interaction terms between specialty and holistic review, was not significant for the outcome of the percentage of female residents (interaction p-value = 0.280) (Table 5). Additionally, there was no evidence of interaction between holistic review and the percentage of female faculty on the outcome of the percentage of female residents on the interaction plot (Figure 1). The marginal slopes were 0.5 (95% CI 0.2 to 0.8) in programs not performing and 0.5 (95% CI 0.3 to 0.6) in programs performing holistic review. Furthermore, the interaction term between holistic review and the percentage of female faculty on the primary outcome (percentage of female residents) was not significant in the full model (coefficient of -0.0%, 95% CI -0.4 to 0.3). The likelihood ratio test between the full and reduced interaction models was not significant (interaction p-value = 0.851) (Table 6). Lastly, the interaction results were similar after multiple imputation of missing values (Tables 7, 8, Figure 2).

**Table 5:** Models for interaction between specialty and holistic review for the outcome of percent female resident physicians ^a^Interaction term The likelihood ratio test between the full and reduced interaction models was not significant (interaction p-value = 0.280) CI, confidence interval

	Full Model	Reduced Model
Variable	Estimate (95% CI)	Estimate (95% CI)
Holistic review: Yes vs. No	-9.8 (-24 to 4.4)	-1.6 (-6.4 to 3.1)
Specialty		
Anesthesiology	Reference	Reference
Emergency medicine	6.1 (-10.5 to 22.8)	7.9 (-0.2 to 16)
Family medicine	24.5 (8.6 to 40.4)	23.8 (16 to 31.7)
Internal medicine	-0.7 (-16.1 to 14.7)	7 (-1.6 to 15.6)
Neurology	6 (-16 to 28.1)	17.4 (7.5 to 27.3)
Obstetrics and gynecology	42 (24.4 to 59.7)	52.1 (43 to 61.2)
Orthopedic surgery	-12.6 (-41.5 to 16.2)	-12.9 (-24.4 to -1.5)
Pediatrics	33 (15.3 to 50.7)	40 (31.2 to 48.8)
Psychiatry	-14.3 (-36.3 to 7.7)	11.4 (1.9 to 20.9)
Surgery (general)	11 (-8.2 to 30.2)	16.6 (7.3 to 26)
Emergency medicine: Holistic review (Yes)^a^	3.6 (-15.5 to 22.7)	Excluded
Family medicine: Holistic review (Yes)^a^	0.8 (-17.6 to 19.2)	Excluded
Internal medicine: Holistic review (Yes)^a^	11.2 (-7.3 to 29.8)	Excluded
Neurology: Holistic review (Yes)^a^	14.9 (-9.8 to 39.7)	Excluded
Obstetrics and gynecology: Holistic review (Yes)^a^	13.9 (-6.7 to 34.5)	Excluded
Orthopedic surgery: Holistic review (Yes)^a^	1.4 (-30.1 to 33)	Excluded
Pediatrics: Holistic review (Yes)^a^	9.9 (-10.5 to 30.2)	Excluded
Psychiatry: Holistic review (Yes)^a^	31.4 (6.8 to 55.9)	Excluded
Surgery (general): Holistic review (Yes)^a^	8.2 (-13.9 to 30.2)	Excluded
Degrees of freedom	183	192
Adjusted R^2^	0.584	0.582

**Table 6:** Models for interaction between the percent female faculty and holistic review for the outcome of percent female resident physicians The likelihood ratio test between the full and reduced interaction models was not significant (interaction p-value = 0.851) CI, confidence interval

	Full Model	Reduced Model
Variable	Estimate (95% CI)	Estimate (95% CI)
Percent female faculty	0.5 (0.2 to 0.8)	0.5 (0.4 to 0.6)
Holistic review: Yes vs. No	0.6 (-15.4 to 16.7)	-0.7 (-7.3 to 5.8)
Female faculty: Holistic review interaction	0.0 (-0.4 to 0.3)	Excluded
Degrees of freedom	187	188
Adjusted R^2^	0.209	0.213

**Table 7:** Models for interaction between specialty and holistic review for the outcome of percent female resident physicians after multiple imputation ^a^Interaction term The likelihood ratio test between the full and reduced interaction models was not significant (interaction p-value = 0.527) CI, confidence interval

	Full Model	Reduced Model
Variable	Estimate (95% CI)	Estimate (95% CI)
Holistic Review: Yes vs. No	-9.6 (-23 to 3.8)	-1.8 (-6.1 to 2.6)
Specialty		
Anesthesiology	Reference	Reference
Emergency medicine	6.1 (-10 to 22.3)	8.4 (1 to 15.8)
Family medicine	21.8 (6.8 to 36.7)	23.1 (16.1 to 30.2)
Internal medicine	0.1 (-14.5 to 14.7)	7.8 (-0.1 to 15.6)
Neurology	6 (-15.3 to 27.4)	17.6 (8.3 to 26.8)
Obstetrics and gynecology	42 (24.9 to 59.2)	52.3 (43.8 to 60.8)
Orthopedic surgery	-12.1 (-33.5 to 9.3)	-12 (-22.4 to -1.6)
Pediatrics	33 (15.9 to 50.1)	40.1 (32 to 48.3)
Psychiatry	-5.5 (-24.2 to 13.2)	11.8 (3.3 to 20.3)
Surgery (general)	11 (-7.6 to 29.7)	16.6 (8 to 25.3)
Emergency medicine: Holistic review (Yes)^a^	3.9 (-14.4 to 22.1)	Excluded
Family medicine: Holistic review (Yes)^a^	2.8 (-14.2 to 19.8)	Excluded
Internal medicine: Holistic review (Yes)^a^	10.8 (-6.6 to 28.1)	Excluded
Neurology: Holistic review (Yes)^a^	14.8 (-9 to 38.6)	Excluded
Obstetrics and Gynecology: Holistic review (Yes)^a^	13.8 (-6 to 33.5)	Excluded
Orthopedic Surgery: Holistic review (Yes)^a^	0.7 (-23.8 to 25.2)	Excluded
Pediatrics: Holistic review (Yes)^a^	9.7 (-9.9 to 29.2)	Excluded
Psychiatry: Holistic review (Yes)^a^	21.8 (0.8 to 42.8)	Excluded
Surgery (general): Holistic review (Yes)^a^	7.8 (-13.3 to 28.9)	Excluded
Degrees of freedom	202	211
Adjusted R^2^	0.580	0.583

**Table 8:** Models for interaction between the percent female faculty and holistic review for the outcome of percent female resident physicians after multiple imputations The likelihood ratio test between the full and reduced interaction models was not significant (interaction p-value = 0.779) CI, confidence interval

	Full Model	Reduced Model
Variable	Estimate (95% CI)	Estimate (95% CI)
Percent female faculty	0.5 (0.2 to 0.8)	0.5 (0.4 to 0.6)
Holistic review: Yes vs. No	0.9 (-13.4 to 15.2)	-0.9 (-6.8 to 5)
Female faculty: Holistic review interaction	0 (-0.3 to 0.3)	Excluded
Degrees of freedom	218	219
Adjusted R^2^	0.216	0.219

## Discussion

A self-reported holistic review of residency applications among the residency programs responding to our survey was not associated with the percentage of female residents at their programs in any of our primary, sensitivity, subgroup, or interaction analyses. However, we consider our results hypothesis-generating because they must be interpreted, considering the possibility of response bias. For example, programs with an applicant selection process to maintain the sex diversity of their matriculating residents, other than holistic review, may have favored the null result. Furthermore, prior work on holistic review in US postgraduate training programs has typically examined its effects on the matriculation of underrepresented minority resident physicians [20-22]. Therefore, holistic review may not be a mechanism that programs use to ensure the sex diversity of their matriculating resident physicians. Lastly, despite the variability in female representation within postgraduate training programs between specialties [16], the overall proportions of male and female applicants in the NRMP match are balanced [28].

Female resident representation in a specialty and within individual programs is likely related to female faculty representation. Prior work indicates an association between the percentage of female faculty and female residents [16]. Similarly, in our reduced model examining the association between the percentage of female faculty and the percentage of female residents adjusting for holistic review, we observed a 0.5% (95% CI 0.4 to 0.6) increase in female residents with a 1% increase in female faculty (Table 6). However, we did not observe an interaction between holistic review and the percentage of female faculty on the percentage of female residents (Figures 1, 2) (Tables 6, 8). This may be due to the fact that female faculty are likely underrepresented in our sample of responding programs, as only approximately 20% of faculty were female in our sample (Table 1), despite approximately 37% of practicing physicians being female [2].

Although definitive conclusions cannot be drawn, we did not observe an association between holistic review and the percentage of female residents after accounting for specialty (Tables 3-5, 7). Although 79% of programs cite increasing resident diversity as a driver for engaging in holistic review [19], holistic review may not be intended to improve sex diversity, even in specialties with overrepresentation of one sex (Tables 3, 4) [16]. However, additional investigation with a more representative sample is necessary to draw more accurate and reliable conclusions.

Lastly, we observed some unexpected findings regarding characteristics not associated with holistic review. For example, approximately three-fourths of the programs in both the holistic review and no holistic review groups still had minimum board examination scores reported (Table 2). This result was unexpected because the use of USMLE cutoffs by programs may worsen diversity [29]. Therefore, this finding suggests that programs may not feel that the use of application filters precludes performing holistic review. This finding is consistent with the 2022 NRMP Program Director Survey, which reported that approximately 40% of applications are rejected by standardized screening without holistic review [19]. Holistic review may be considered a post-screening process rather than a process that is implemented for every application. This is likely because holistic review of every application to a program may be unfeasible for many programs, given the rising overapplication by applicants [30], which further incentivizes the use of filters [26]. Additional investigations are needed to understand the potential interaction between application filters and holistic review on matriculating resident diversity.

Our study has some limitations. First, the response rate of 6.6% limits the generalizability of the results, and we did not attempt any methods (e.g., weighting of items or propensity scores) to adjust for the nonrepresentative sample, given the low response rate. Therefore, we cannot comment on the majority of programs and only infer that our analysis applies to the sample of respondents. Similarly, programs report data to FREIDA, exposing the results to information bias. Nevertheless, the program characteristics between responders and nonresponders were similar (Table 1).

Additionally, the results do not apply beyond the 10 specialties that we examined. We did not stratify analyses by individual specialties due to the limited number of respondents within most specialties. Therefore, the results suggest that holistic review may not be associated with female resident representation overall. Still, they cannot be used to draw any conclusions regarding the association between holistic review and female resident representation within a given specialty, program type, or other subgroup. Similarly, holistic review processes are tailored to each program’s recruitment objectives [20-22]; therefore, holistic review may benefit individual programs with regard to their individual goals [20-22] without a population-level effect for any one outcome (e.g., percentage of female residents).

Furthermore, several variables, including the percentage of female residents and faculty, had missing values. However, the degree of missingness was <10%, and we repeated the analyses after multiple imputations, which did not reveal any differences in the examined outcomes. Nevertheless, multiple imputation assumes that imputed missing values are missing at random and are related to the observed variables used in the dataset. It was unknown when holistic review may have been implemented within each program. Therefore, we did not know if holistic review was performed when the current residents applied to their programs. This may have resulted in a misclassification bias. We used a single definition of holistic review; therefore, some programs that conduct their version of holistic review may not have been classified as conducting holistic review if it does not fit our definition. We did not account for the sex diversity of patients within each specialty. For example, if the goal is concordant proportions of female patients to female physicians, then the workforce sex diversity is expected to differ between specialties (e.g., urology vs. gynecology).

Similarly, we could not account for the sex diversity of the applicant pool in each specific program. Despite the best efforts of a program to fairly evaluate all applicants, programs may have less control over the sex diversity of their applicant pool beyond recruiting. Lastly, we reported female representation according to the FREIDA data, and it is unclear if female refers to sex or gender in the data. As per the Sex and Gender Equity in Research (SAGER) guidelines, male and female refers to an individual’s sex, not gender (e.g., man, woman, transgender, nonbinary) [31]. Best practices would be to collect self-reported sex and gender information from each individual physician and subsequently aggregate the sex and gender variables to the program level. Therefore, it is possible that our previous assumption regarding the male and female sex representation at the program level may be fully or in part related to gender.

## Conclusions

In our multispecialty sample, holistic review of residency applications was not associated with the percentage of female residents, nor did it modify the association between female faculty and resident representation. Additionally, approximately three-fourths of the programs in both groups reported minimum board examination scores. Further work is needed to understand the use and efficacy of holistic review in addressing the imbalance of male and female physicians in the healthcare workforce, particularly between specialties.

## Figures and Tables

**Figure 3:**
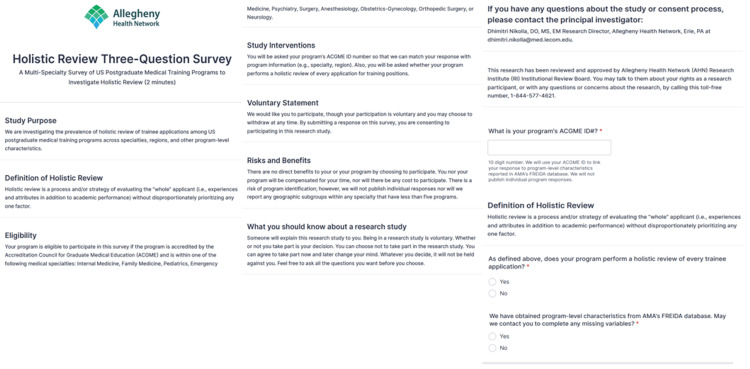
Holistic review three-question survey

## Appendices

**Table 9:** Completed checklist for reporting results of the Internet E-surveys (CHERRIES)

Checklist Item	Explanation	Location
Describe survey design	Describe target population and sample frame. Is the sample a convenience sample? (In “open” surveys, this is most likely).	Methods – Sample Characteristics
IRB approval	Mention whether the study has been approved by an IRB.	Methods – Survey Preparation and Ethical Considerations
Informed consent	Describe the informed consent process. Where were the participants told the length of time of the survey, which data were stored and where and for how long, who the investigator was, and the purpose of the study?	Methods – Survey Preparation and Ethical Considerations
Data protection	If any personal information was collected or stored, describe what mechanisms were used to protect unauthorized access.	Methods – Survey Preparation and Ethical Considerations
Development and testing	State how the survey was developed, including whether the usability and technical functionality of the electronic questionnaire had been tested before fielding the questionnaire.	Methods – Survey Administration
Open survey vs. closed survey	An “open survey” is a survey open for each visitor of a site, while a closed survey is only open to a sample that the investigator knows (password-protected survey).	Methods – Survey Administration
Contact mode	Indicate whether or not the initial contact with the potential participants was made on the Internet (Investigators may also send out questionnaires by mail and allow for a web-based data entry).	Methods – Survey Administration
Advertising the survey	How/where was the survey announced or advertised? Some examples are offline media (newspapers), or online (mailing lists – If yes, which ones?) or banner ads (Where were these banner ads posted and what did they look like?). It is important to know the wording of the announcement as it will heavily influence who chooses to participate. Ideally, the survey announcement should be published as an appendix.	N/A – Emailed directly to program directors
Web/E-mail	State the type of e-survey (e.g., one posted on a website, or one sent out through e-mail). If it is an e-mail survey, were the responses entered manually into a database, or was there an automatic method for capturing responses?	Methods – Survey Administration
Context	Describe the website (for mailing list/newsgroup) in which the survey was posted. What is the website about, who is visiting it, what are visitors normally looking for? Discuss to what degree the content of the website could preselect the sample or influence the results. For example, a survey about vaccination on a anti-immunization website will have different results from a web survey conducted on a government website.	N/A – The web address linked directly to the Jotform survey
Mandatory/voluntary	Was it a mandatory survey to be filled in by every visitor who wanted to enter the website, or was it a voluntary survey?	Methods – Survey Preparation and Ethical Considerations
Incentives	Were any incentives offered (e.g., monetary, prizes, or nonmonetary incentives such as an offer to provide the survey results)?	Methods – Survey Preparation and Ethical Considerations
Time/date	In what timeframe were the data collected?	Methods – Survey Administration
Randomization of items or questionnaires	To prevent biases items can be randomized or alternated.	Methods – Survey Administration
Adaptive questioning	Use adaptive questioning (certain items, or only conditionally displayed based on responses to other items) to reduce number and complexity of the questions.	N/A
Number of Items	What was the number of questionnaire items per page? The number of items is an important factor for the completion rate.	Methods – Survey Administration
Number of screens (pages)	Over how many pages was the questionnaire distributed? The number of items is an important factor for the completion rate.	Methods – Survey Preparation and Ethical Considerations
Completeness check	It is technically possible to do consistency or completeness checks before the questionnaire is submitted. Was this done, and if “yes,” how (usually JAVAScript)? An alternative is to check for completeness after the questionnaire has been submitted (and highlight mandatory items). If this has been done, it should be reported. All items should provide a nonresponse option such as “not applicable” or “rather not say,” and the selection of one response option should be enforced.	Methods – Missingness
Review step	State whether respondents were able to review and change their answers (e.g., through a Back button or a Review step that displays a summary of the responses and asks the respondents if they are correct).	Methods – Survey Preparation and Ethical Considerations
Unique site visitor	If you provide view rates or participation rates, you need to define how you determined a unique visitor. There are different techniques available, based on IP addresses or cookies or both.	N/A
View rate (ratio of unique survey visitors/unique site visitors)	Requires counting unique visitors to the first page of the survey, divided by the number of unique site visitors (not page views!). It is not unusual to have view rates of less than 0.1 % if the survey is voluntary.	N/A
Participation rate (ratio of unique visitors who agreed to participate/unique first survey page visitors)	Count the unique number of people who filled in the first survey page (or agreed to participate, e.g., by checking a checkbox), divided by visitors who visit the first page of the survey (or the informed consents page, if present). This can also be called “recruitment” rate.	Methods – Survey Administration
Completion rate (ratio of users who finished the survey/users who agreed to participate)	The number of people submitting the last questionnaire page, divided by the number of people who agreed to participate (or submitted the first survey page). This is only relevant if there is a separate “informed consent” page or if the survey goes over several pages. This is a measure for attrition. Note that “completion” can involve leaving questionnaire items blank. This is not a measure for how completely questionnaires were filled in. (If you need a measure for this, use the word “completeness rate”).	Methods – Survey Administration
Cookies used	Indicate whether cookies were used to assign a unique user identifier to each client computer. If so, mention the page on which the cookie was set and read, and how long the cookie was valid. Were duplicate entries avoided by preventing users access to the survey twice; or were duplicate database entries having the same user ID eliminated before analysis? In the latter case, which entries were kept for analysis (e.g., the first entry or the most recent)?	Methods – Survey Administration
IP check	Indicate whether the IP address of the client computer was used to identify potential duplicate entries from the same user. If so, mention the period of time for which no two entries from the same IP address were allowed (e.g., 24 hours). Were duplicate entries avoided by preventing users with the same IP address access to the survey twice; or were duplicate database entries having the same IP address within a given period of time eliminated before analysis? If the latter, which entries were kept for analysis (e.g., the first entry or the most recent)?	Methods – Survey Administration
Log file analysis	Indicate whether other techniques to analyze the log file for identification of multiple entries were used. If so, please describe.	Methods – Survey Administration
Registration	In “closed” (non-open) surveys, users need to login first, and it is easier to prevent duplicate entries from the same user. Describe how this was done. For example, was the survey never displayed a second time once the user had filled it in, or was the username stored together with the survey results and later eliminated? If the latter, which entries were kept for analysis (e.g., the first entry or the most recent)?	N/A
Handling of incomplete questionnaires	Were only completed questionnaires analyzed? Were questionnaires which terminated early (where, e.g., users did not go through all questionnaire pages) also analyzed?	Methods – Missingness
Questionnaires submitted with an atypical timestamp	Some investigators may measure the time people needed to fill in a questionnaire and exclude questionnaires that were submitted too soon. Specify the timeframe that was used as a cutoff point, and describe how this point was determined.	Methods – Survey Administration
Statistical correction	Indicate whether any methods such as weighting of items or propensity scores have been used to adjust for the nonrepresentative sample; if so, please describe the methods.	Discussion – Limitations

**Table 10:** Coding of the study variables COMLEX-USA, Comprehensive Osteopathic Medical Licensing Examination; DO, doctor of osteopathic medicine; IMG, international medical graduate; FREIDA, Fellowship and Residency Electronic Interactive Database Access; USMLE, United States Medical Licensing Examination

Variable	Collected	Coded
Holistic review	Binary (yes/no) from the survey.	Binary (yes/no) – No modification
Program type	Categorical as either University Hospital, Community Hospital, Military, Other, or Community-based university-affiliated hospital from FRIEDA.	Categorical (same values) – No modification
Specialty	Categorical as either anesthesiology emergency medicine, family medicine, internal medicine, neurology, obstetrics and gynecology, orthopedic surgery, pediatrics, psychiatry, or surgery-general from FRIEDA.	Categorical (same values) – No modification
Minimum board examination score	Derived from minimum scores on USMLE Steps 1 and 2 and COMLEX-USA Levels 1 and 2, reported by the programs in FREIDA. If a minimum score on either test was reported, then the program was coded as requiring a minimum score.	Binary (yes/no)
USMLE required for DOs	Derived COMLEX-USA Levels 1 and 2 required fields in FREIDA. If the program reported that the corresponding USMLE Step score was required in either of these fields, then the program was coded as requiring the USMLE for DOs.	Binary (yes/no)
J1 visa sponsored	Binary (yes/no) from FREIDA.	Binary (yes/no) – No modification
H1B visa sponsored	Binary (yes/no) from FREIDA.	Binary (yes/no) – No modification
F1 visa sponsored	Binary (yes/no) from FREIDA.	Binary (yes/no) – No modification
Osteopathic recognition	Binary (yes/no) from FREIDA.	Binary (yes/no) – No modification
Percent DO residents	Continuous as the 3-year average percentage of DO resident physicians in the program including the 2021-2022 academic year from FREIDA.	Continuous – No modification
Percent IMG residents	Continuous as the 3-year average percentage of IMG resident physicians in the program including the 2021-2022 academic year from FREIDA.	Continuous – No modification
Percent female residents	Continuous as the 3-year average percentage of female resident physicians in the program including the 2021-2022 academic year from FREIDA.	Continuous – No modification
Percent female faculty	Continuous as the percentage of full-time female faculty physicians in the program from FREIDA.	Continuous – No modification
First-year positions	Continuous, the count of available spots in the first year of the program from FREIDA.	Continuous – No modification

**Table 11:** Missingness for model variables before and after multiple imputation ^a^The denominator was 222, the total number of programs that responded Note: Since the mechanism for missingness cannot be definitively determined and the primary assumption of multiple imputation is that the missing values are missing at random, we included as many predictor variables as possible, including variables not examined in the study. Variables used for multiple imputation included minimum board examination score, USMLE required for DOs, visa sponsored (J1, H1B, F1), meal allowance, osteopathic recognition, onsite childcare, subsidized childcare, housing stipend, specialty, program type, number of first-year positions, percent DO residents, percent IMG residents, first-year salary, days of paid medical leave, days of unpaid medical leave, paid vacation days, and paid sick days. We used random forest multiple imputation with 10 iterations and 100 trees. The out-of-bag error was 0.06879093, and the proportion of falsely classified was 0.09196338

Model Variables	Missingness Before Multiple Imputation, N (%)^a^	Missingness After Multiple Imputation, N (%)
Holistic review	0 (0.0)	0 (0.0)
Specialty	0 (0.0)	0 (0.0)
Percent female residents	19 (8.6)	0 (0.0)
Percent female faculty	16 (7.2)	0 (0.0)
